# Evaluation of volumetric modulated arc therapy for postmastectomy treatment

**DOI:** 10.1186/1748-717X-9-66

**Published:** 2014-02-26

**Authors:** Geoffrey P Nichols, Jonas D Fontenot, John P Gibbons, Mary Ella Sanders

**Affiliations:** 1 Department of Physics and Astronomy, Louisiana State University and Agricultural & Mechanical College, Baton Rouge, LA, USA; 2 Mary Bird Perkins Cancer Center, Baton Rouge, LA 70809, USA

**Keywords:** Volumetric modulated arc therapy, Helical tomotherapy, Post mastectomy

## Abstract

**Purpose:**

To examine the feasibility of volumetric modulated arc therapy (VMAT) for post mastectomy radiotherapy (PMRT).

**Methods and materials:**

Fifteen PMRT patients previously treated at our clinic with helical tomotherapy (HT) were identified for the study. Planning target volumes (PTV) included the chest wall and regional lymph nodes. A systematic approach to constructing VMAT that met the clinical goals was devised. VMAT plans were then constructed for each patient and compared with HT plans with which they had been treated. The resulting plans were compared on the basis of PTV coverage; dose homogeneity index (DHI) and conformity index (CI); dose to organs at risk (OAR); tumor control probability (TCP), normal tissue complication probability (NTCP) and secondary cancer complication probability (SCCP); and treatment delivery time. Differences were tested for significance using the paired Student’s t-test.

**Results:**

Both modalities produced clinically acceptable PMRT plans. VMAT plans showed better CI (*p* < 0.01) and better OAR sparing at low doses than HT plans, particularly at doses less than 5 Gy. On the other hand, HT plans showed better DHI (*p* < 0.01) and showed better OAR sparing at higher doses. Both modalities achieved nearly 100% tumor control probability and approximately 1% NTCP in the lungs and heart. VMAT showed lower SCCP than HT (*p* < 0.01), though both plans showed higher SCCP values than conventional mixed beam (electron-photon) plans reported by our group previously. VMAT plans required 66.2% less time to deliver than HT.

**Conclusions:**

Both VMAT and HT provide acceptable treatment plans for PMRT. Both techniques are currently utilized at our institution.

## Introduction

Post-mastectomy radiotherapy (PMRT) presents a challenging treatment geometry. The target volume - typically consisting of the chest wall (CW) and regional lymph nodes - covers a large, superficial area that is thin and convex in shape, and is immediately adjacent to the lung, heart, and contralateral breast. A variety of techniques have been proposed for PMRT. In our clinic, PMRT was historically treated with a mixed-beam technique consisting of anterior electrons to treat the medial CW and internal mammary nodes (IMN), oblique electrons to treat the lateral CW, and parallel-opposed x-ray to treat the supraclavicular (SC) and axillary (AX) nodes. Because of the need to junction fields, edge feathering was typically utilized to reduce heterogeneities to acceptable levels; however, residual heterogeneities were inevitable and treatment setup times were lengthy and laborious. Subsequently, our group previously reported on the use of helical tomotherapy (HT) for PMRT [1] to improve ease of setup and dose conformity and homogeneity of PMRT treatments, albeit at the cost of larger volumes of normal tissue receiving doses less than 25 Gy. Nonetheless, our previous findings resulted in HT often being the treatment of choice for PMRT in our clinic. However, the use of HT has limitations, including limited availability of and access to the technology in addition to the larger low dose volumes noted above.

Volumetric modulated arc therapy (VMAT) is a rotational IMRT delivery technique that is widely available on most contemporary linear accelerators. VMAT has been shown to provide dose distributions comparable to fixed-beam IMRT while improving treatment efficiency for a variety of cases [2-5], including PMRT [6,7].

However, for PMRT, those studies compared VMAT with conventional 3D conformal techniques only and, in particular, there are currently no known studies that have compared VMAT with HT for PMRT. Since studies comparing VMAT and HT for other sites have produced mixed results [2,8,9], it was unknown if VMAT could complement HT as a competitive alternative in our PMRT program.

The objectives of this study were to (1) devise a systematic method of constructing VMAT plans to meet our clinical goals, similar to our previous approach with HT and (2) compare VMAT planning results with HT plans for patients previously treated for PMRT in our clinic. VMAT plans were constructed for 15 PMRT patients previously treated with HT. The resulting plans were compared based on dosimetric quality, radiobiological calculations and delivery efficiency.

## Methods and materials

### Patients

A power analysis of data from previous studies (8, 9) indicated a required study size of fifteen patients, who were randomly selected from a list of PMRT patients previously treated with HT in our clinic: 7 requiring irradiation of the right CW, 8 requiring irradiation of the left CW. 11 patients in the study underwent unilateral mastectomy on the side of irradiation, with the remaining 4 having bilateral mastectomy (though those patients were only irradiated on one side). Each patient’s CT data set was anonymized and placed in database compliant with the Health Insurance Portability and Accountability Act. Research ethics was obtained from the institutional research policy committee. The average age of the patient cohort was 60.5 years (range: 30 to 85 years).

### Simulation, contouring, and planning goals

All patients underwent three-dimensional treatment simulation in the supine position with a custom 1-cm thick solid thermoplastic bolus (Aquaplast RT®, Radiation Products Design, Inc., Albertville, MN) added to the CW daily to increase the skin dose and to assist in creating flash during treatment planning. The planning target volume was delineated by a radiation oncologist, and included the CW (including bolus) and relevant nodal regions (IMN, AX, and SCV). Delineated organs at risk (OAR) included the lungs, heart, contralateral breast, esophagus, and spinal cord.

The prescription dose for all patients was 50.4 Gy in 28 fractions. Dosimetric planning goals (see Table 1) were determined from normal tissue tolerances by Emami *et al.*[10] and from the clinical experience of radiation oncologists at our clinic.

**Table 1:** Dosimetric planning goals for the PMRT patients in this study

**Structure**	**Dose**	**Volume**
PTV	< 55 Gy	1%
	50.4 Gy	90%
	> 46 Gy	99%
Lungs	< 20 Gy	18%
Heart	< 20 Gy	18%
Breast	< 5 Gy	1%
Liver	< 15 Gy	15%
Airway	< 30 Gy	1%
Esophagus	< 30 Gy	1%
Cord	< 25 Gy	1%
Normal Tissue	< 55 Gy	1%

Due to the potentially subjective nature of treatment planning, extreme care was taken to develop a systematic and rigorous procedure for producing the optimum plan for each patient and modality. The planning variables for each delivery approach were carefully investigated and their impact on plan quality was documented. The approaches to producing HT and VMAT treatment plans for each patient are described below.

### Helical tomotherapy planning

HT treatment plans were created using the TomoTherapy treatment planning system (version 3.1.2) with treatment values typical of clinical delivery in our clinic, including a nominal jaw width of 5 cm, a pitch of 0.287, and a “normal” grid size. HT plans were optimized with a maximum modulation factor of 3.0 in “beamlet” mode, which performs an initial full dose calculation to determine the contribution of dose from every possible leaf opening, then optimizes leaf open times to minimize the optimization objective function. Initial optimization objectives were set to achieve the planning goals, but adjusted during optimization to produce the best plan for each patient using a systematic procedure described in detail elsewhere [11]. A minimum of 250 total optimization iterations were completed until planning objectives were met or until the plan could no longer be improved.

### Volumetric modulated arc therapy planning

VMAT treatment plans were created using the Philips Pinnacle treatment planning system (version 9.0, Philips Medical Systems, Fitchburg, WI). All VMAT treatment plans were constructed for an Elekta Infinity radiotherapy system (Elekta AB, Stockholm, Sweden) using 6 MV photons. All patients were planned using a couch angle of 0° and collimator angle of 45°. Two partial VMAT arcs of 220° each were utilized, with the start and stop angles of the first arc set to 50° and 190° (IEC convention, rotating counterclockwise), respectively, for right CW patients and 170° and 310° (rotating clockwise), respectively, for left CW patients. These angles were chosen to avoid direct irradiation of the spinal cord, contralateral breast and contralateral lung during irradiation. The dose grid was set to the default value of 4 × 4 × 4 mm^3^. Optimization was performed using the SmartArc module using a 4 degree final gantry spacing, a leaf motion constraint of 4 mm per degree of arc rotation, and a maximum delivery time of 60 seconds per 220° partial arc. Initial optimization objectives were set to achieve the planning goals, but adjusted during optimization using a systematic procedure found to produce optimal plan quality. Additional details of the VMAT planning procedure can be found elsewhere [11].

### Plan comparison metrics

HT and VMAT plans were compared on the basis of dosimetric end points, radiobiological calculations, and delivery efficiency. The following quantities were compared: target coverage (D_95%_), dose homogeneity, conformity, and tumor control probability (TCP) for the PTV; irradiated volumes, normal tissue complication probability (NTCP), and secondary cancer complication probability (SCCP) for the normal tissues.

In the PTV, the dose homogeneity was compared using the dose homogeneity index (DHI), calculated as

$$\mathrm{DHI}=\frac{D_{\text{max}}-D_{\text{min}}}{D_{\mathrm{Rx}}}$$

where *D*_max_, *D*_min_, and *D*_Rx_ denote the maximum, minimum, and prescription doses, respectively. Values of DHI are unitless, with smaller values representing more uniform doses. Dose conformity was compared using the conformity index (CI), calculated as

$$\mathrm{CI}=\frac{TV_{\mathrm{PIV}}}{\mathrm{TV}}\times \frac{TV_{\mathrm{PIV}}}{\mathrm{PIV}}$$

where *TV* is the target volume, *PIV* is the volume of the prescribed isodose value and *TV*_*PIV*_ is the volume of the target that is covered by the prescribed isodose value. Values of CI are unitless, with larger values representing better dose conformity. Finally, TCP was calculated using a linear quadratic model for survival fraction with a repair mechanism correction [12]. Values for breast cancer taken from Wigg [13] were α (=0.51 Gy^-1^), β (=0.061 Gy^-2^), repair time (=1 h), and clonogenic cell density (10^7^ cm^-3^).

Doses to the normal tissues were evaluated using fractional volumes consistent with the planning goals and relevant clinical end points. The percent volume of the lungs and heart receiving greater than 20 Gy (V_20Gy_) and 15 Gy (V_15Gy_), respectively, were compared. The mean dose to the contralateral breast was also evaluated.

Normal tissue complication probabilities (NTCP) for radiation-induced pneumonitis were computed for the lung using the Lyman-Kutcher-Berman probit model [14-16] using the following values: D_50_ = 24.5 Gy (10), n = 0.87, and m = 0.18 (16). NTCP for radiation-induced ischema for the heart was calculated using the relative seriality model [17] using the following values: D_50_ = 52.3 Gy, s = 1.0, and γ = 1.28 [18].

The organ equivalent dose (OED) formalism of Schneider [19,20] was used to compute secondary cancer complication probability (SCCP) for the lungs (α = 0.085 Gy^-1^, In_org_ = 1.68% Gy^-1^), contralateral breast (α = 0.085 Gy^-1^, In_org_ = 0.78% Gy^-1^), and normal tissue (excludes delineated PTV and OARs; α = 0.085 Gy^-1^, In_org_ = 1.76% Gy^-1^). In_org_ was estimated using atomic bomb survivor data and applies to whole-body irradiation. Because the CT sets for these patients were limited to partial body scans, the SCCP for normal tissue was reduced by the ratio of the volume of normal tissue to the volume of an average woman, calculated using the average weight, 74.7 kg, of women between the ages of 20 and 74 [21], and an estimated average density of 0.001 kg/cm^3^.

Delivery efficiency was evaluated by comparing the actual treatment delivery time for each plan. The delivery time of each VMAT plan was measured using a stopwatch. HT delivery times were taken from the planning system report, which contains an accurate estimate of delivery time for each patient.

All values (dosimetric, radiobiological, and delivery) from the fifteen patients were averaged and compared. Differences were evaluated for statistical significance (*p* < 0.05) using the Student’s paired *t*-test.

## Results

**Figure 1:**
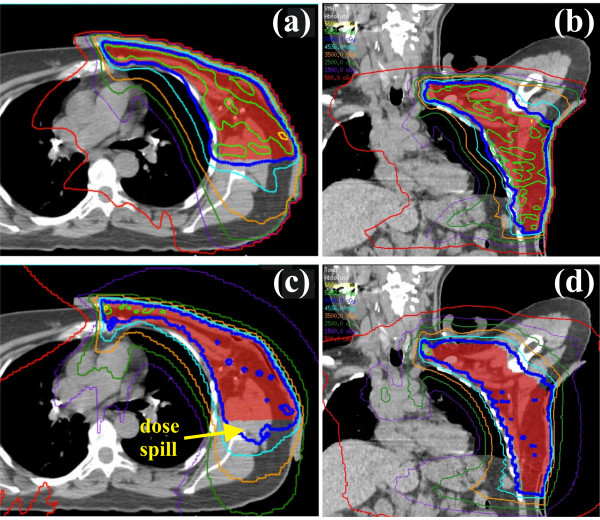
**Comparison of VMAT and HT treatment plans.** Transverse and coronal isodose distributions for the VMAT **(a, b)** and HT plans **(c, d)** are shown. Isodose contours are 55, 52.9 (105%), 50.4, 45.3 (90%), 35, 25, 15, and 5 Gy.

**Figure 2:**
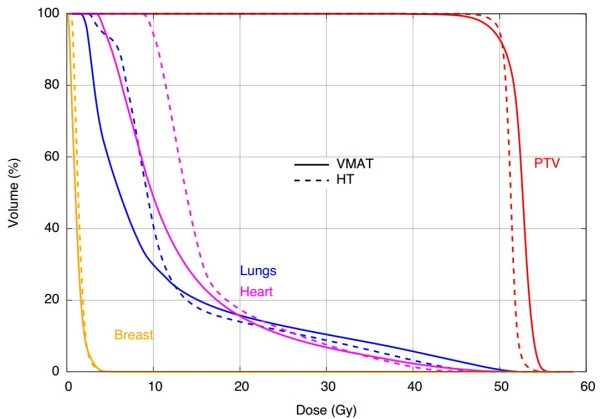
**Comparison of VMAT and HT treatment plans.** Dose-volume histograms for the distributions shown in Figure 1.

Isodose distributions and dose-volume histograms for a representative patient are shown in Figures 1 and 2, respectively. The figure shown compares a VMAT and HT plan in the axial and coronal planes. The VMAT plan contained larger contiguous regions of 105% of the prescription dose within the PTV compared with the HT plan, indicating better homogeneity with HT (DHI = 0.161 and 0.085 for VMAT and HT, respectively). Comparable PTV coverage was noted between the plans, with HT failing to cover the edge of the PTV near the medial aspect of the lung. The VMAT plan showed better dose conformity (CI = 0.773 and 0.700 for VMAT and HT, respectively), with the HT plan consistently showing prescription doses in normal tissue located lateroposterior to the PTV. Lower isodose lines (*i.e.*, 25, 15, and 5 Gy) also extended further into normal tissue in the HT plan.

Table 2 compares the results of the VMAT and HT plans with for a variety of dosimetric and radiobiological metrics. Values are presented as means for the fifteen patients. In general, differences between the delivery approaches were small but statistically significant, indicating a consistent advantage (or disadvantage) in each technique across the patient cohort. These trends are discussed in more detail below.

**Table 2:** Summary comparison of VMAT and HT plans

**Structure/Item**	**Metric**	**VMAT**	**HT**	***p*-value**
PTV	D_V95%_ (Gy)	49.3 ± 0.1	49.8 ± 0.1	<0.001
	DHI	0.147 ± 0.009	0.096 ± 0.005	<0.001
	CI	0.778 ± 0.008	0.719 ± 0.008	<0.001
	TCP	99.8 ± 0.2	100 ± 0.0	0.002
Total lung	V_5Gy_ (%)	66.2 ± 3.1	89.8 ± 1.4	<0.001
	V_20Gy_ (%)	16.3 ± 0.2	15.0 ± 0.3	<0.001
	NTCP (%)	0.3 ± 0.1	0.6 ± 0.1	0.06
	SCCP (%)	5.3 ± 0.1	6.1 ± 0.0**	<0.001
Ipsilateral lung	V_5Gy_ (%)	96.9 ± 1.3	99.3 ± 0.5	0.06
	V_20Gy_ (%)	32.3 ± 0.8	29.9 ± 1.0	<0.001
Contralateral lung	V_5Gy_ (%)	37.8 ± 4.9	81.7 ± 2.5	<0.001
Heart*	V_15Gy_ (%)	26.0 ± 1.9	28.2 ± 2.6	0.4
	D_mean_ (Gy)	12.9 ± 0.5	14.4 ± 0.6	0.04
	D_max_ (Gy)	42.2 ± 1.6	38.3 ± 1.3	<0.001
	NTCP (%)	1.3 ± 0.2	0.9 ± 0.1	0.008
Contralateral breast	Mean dose (Gy)	1.5 ± 0.1	1.8 ± 0.1	<0.001
	SCCP (%)	1.0 ± 0.0**	1.2 ± 0.0**	<0.001
Non-specific normal tissue	Mean dose (Gy)	7.0 ± 0.2	11.1 ± 0.4	<0.001
	SCCP (%)	0.7 ± 0.0	0.9 ± 0.0**	<0.001
Efficiency	Delivery time (s)	128.6 ± 2.8	382.4 ± 10.9	<0.001

Values shown are averages of the 15 patients (± 1σ) with the exception of the heart.

*Patients with right-side mastectomy excluded.

**Standard deviation of the mean < 0.05.

*Abbreviations: PTV* planning target volume, *DHI* dose homogeneity index (ideal = 0), *CI* conformity index (ideal = 1), *TCP* tumor control probability, *NTCP* normal tissue complication probability, *SCCP* secondary cancer complication probability.

### Planning target volume

Both VMAT and HT provided clinically acceptable doses to the PTV, though significant (*p* < 0.05) differences were noted between the plans. For example, HT plans consistently showed slightly better coverage ($\bar{D_{V95\%}}=49.8\;\mathrm{Gy}$*vs.* 49.3 Gy, *p* < 0.001) and better dose homogeneity ($\bar{\mathrm{DHI}}=0.096$*vs.* 0.147, *p* < 0.001) in PTV. On the other hand, VMAT showed better dose conformity ($\bar{\mathrm{CI}}=0.778$*vs.* 0.719, *p* < 0.001), primarily owing to the consistent dose spill beyond the posterior region of the PTV in HT plans. For radiobiological calculations, both modalities achieved nearly 100% TCP.

The quality of the high region of dose distribution of VMAT plans was influenced by whether the patient underwent unilateral or bilateral mastectomy despite the fact that all patients underwent radiotherapy planning for unilateral irradiation. Table 3 shows PTV metrics for VMAT and HT plans for each subpopulation. VMAT dose distributions were significantly more homogeneous (*p* < 0.05) and conformal in patients who underwent bilateral mastectomy compared with those who did not. Such differences were not observed in HT plans, nor were differences in normal tissue sparing noted for either modality between the two subpopulations.

**Table 3:** Summary of VMAT and HT plans comparing patients who underwent unilateral and bilateral mastectomy

**Modality**	**Metric**	**Unilateral mastectomy**	**Bilateral mastectomy**	***p*-value**
VMAT	D_V95%_ (Gy)	49.2 ± 0.1	49.6 ± 0.1	<0.001
	DHI	0.161 ± 0.006	0.106 ± 0.014	0.001
	CI	0.766 ± 0.007	0.811 ± 0.012	0.004
	TCP (%)	99.8 ± 0.0*	99.8 ± 0.1	0.4
HT	D_V95%_ (Gy)	49.8 ± 0.1	50.0 ± 0.1	0.4
	DHI	0.101 ± 0.007	0.082 ± 0.004	0.1
	CI	0.715 ± 0.010	0.731 ± 0.013	0.4
	TCP (%)	100.0 ± 0.0	100.0 ± 0.0	1.0

Values shown are averages of the 15 patients (± 1σ) with the exception of the heart.

*Standard deviation of the mean < 0.05.

*Abbreviations: TCP* tumor control probability, *DHI* dose homogeneity index (ideal = 0), *CI* conformity index (ideal = 1).

### Normal tissues

Both VMAT and HT also produced clinically acceptable normal tissue doses. In general, differences were small but significant (*p* < 0.05) with VMAT showing better dose sparing at low dose intervals (*e.g.*, < 15 Gy) and HT showing better dose sparing at higher doses. In the lung, V_5Gy_ was approximately 25% lower (*p* < 0.001) in VMAT plans, while V_20Gy_ was approximately 9% lower (*p* < 0.001) in HT plans. The NTCP for post-radiation pneumonitis was lower in VMAT plans, but was not significant (*p* = 0.06). The SCCP in the lung from VMAT plans was 5.3 ± 0.1% compared with 6.1 ± 0.0% from HT plans (*p* < 0.001).

In the heart, differences in V_15Gy_ and V_30Gy_ were small and not statistically significant. However, because VMAT, on average, showed a larger V_30Gy_, the NTCP for ischemia was higher in VMAT plans (*p* = 0.008). In the contralateral breast, differences in the mean dose and SCCP were small but were statistically significant (*p* < 0.001) in favor of VMAT plans. Finally, VMAT showed a slightly lower mean dose and SCCP compared with HT (*p* < 0.001), though values of SCCP were less than 1%.

### Delivery efficiency

Delivery time measurements showed that VMAT plans were delivered significantly faster at 128.6 ± 2.8 seconds, compared with 382.4 ± 10.9 seconds (*p* < 0.001) for HT plans. Delivery of all planned treatments was confirmed via quality assurance measurements using a two dimension diode array (MapCHECK2, Sun Nuclear Corp., Melbourne, FL USA), with all plans showing better than 90% of measured dose points within 3% dose difference or 3 mm distance to agreement of planned dose points.

## Discussion

VMAT and HT plans were clinically comparable for PMRT, with VMAT plans needing approximately 66% less time to deliver. HT plans showed significantly better dose homogeneity and sparing of normal tissues at dose intervals greater than approximately 15 Gy. On the other hand VMAT plans showed significantly better dose conformity and better sparing of normal tissues at dose intervals less than approximately 10 Gy.

While HT plans showed better dose homogeneity, differences between HT and VMAT plans were smaller in patients who underwent bilateral mastectomy owing to improved dose homogeneity (and conformity) of VMAT plans in that subpopulation. The reason for this is that the contralateral intact breast presented as a difficult avoidance structure during volumetric optimization of PMRT plans, typically showing the greatest objective value of all normal tissue constraints. This suggests that plan quality can be improved in PMRT patients for whom contralateral breast dose is of less concern (*e.g.*, bilateral mastectomy, patients with advanced age, etc.). In contrast, HT plans - which can be more highly modulated at each helical slice of delivery - did not show this effect.

Differences in radiobiological calculations were small but statistically significant across modalities. Both plans showed nearly 100% tumor control. In normal tissue, both plans predicted only slight risk of lung and cardiac toxicities, with VMAT showing a small advantage for the former and HT showing a small advantage for the latter. HT showed values of SCCP that were significantly higher than that of VMAT plans in the lung (0.9 percentage points), contralateral breast (0.2), and non-specific normal tissues (0.2). However, while VMAT plans showed lower SCCP values in this study, a previous study from our group showed that SCCP values of HT plans were approximately twice that of conventional mixed beam (photon-electron) plans in PMRT patients [1] and, therefore, VMAT plans should show similarly elevated SCCP values if compared with mixed beam plans. It should also be noted that the radiobiological model coefficients have considerable uncertainty, and that absolute values of risk, while small, should be interpreted with caution.

Treatment planning results from this study are comparable to other studies that have investigated HT and VMAT for locoregional irradiation for breast cancer, though none to our knowledge have reported use of the 5 cm jaw width for HT or the specific case of postmastectomy irradiation for VMAT. For VMAT, Popescu *et al.*[22] reported PTV coverage and homogeneity values for node-positive breast patients that are similar to the present study. OAR sparing was similar for the lung (V_20Gy_ = 16.3% *vs.* 16.9%) but slightly better for the heart in their study (D_mean_ of 12.9 Gy *vs.* 10.9 Gy), which may be attributable to a lower prescription dose of 45 Gy to internal mammary nodes. For HT, a previous study from our institution showed acceptable dosimetric results from HT plans using a 2.5 cm nominal jaw width for PMRT planning; however, due to the large treatment area, treatment delivery times exceeded 20 minutes [1], which is consistent with a previous study from Hijal *et al.*[23]. Because of this, use of the 5 cm jaw for PMRT in our clinic has significantly reduced treatment delivery time to about 6 minutes with corresponding degradation of plan quality, primarily in the dose-falloff region between the PTV and OARs (*i.e.*, heart and ipsilateral lung). Despite the change, OAR doses were still within acceptable limits and determined to be worth the substantial reduction in treatment delivery time. With respect to the current study, use of the 2.5 cm jaw would likely have only served to widen the observed dosimetric advantage of HT in the lung, with some additional gains in cardiac sparing, again at the expense of marked increase in delivery time. The advantage in low dose spill (*i.e.*, V_5Gy_ volumes) for VMAT plans would likely not be affected since difference in these dose-volumes between our previous (using the 2.5 cm jaw) and current (using the 5 cm jaw) studies are negligible.

One limitation of the study was that only one dose-volume objective (*e.g.*, V_20Gy_ < 20%) could be specified for a given organ at risk in the TomoTherapy planning system. As a result, it was not feasible to include lower dose objectives to control the low dose spill throughout the irradiated volume of each patient during optimization. However, the latest version of the TomoTherapy planning system now offers the user the ability to input multiple dose-volume objectives for organs at risk, and it is possible that the low dose volume in HT plans could have been reduced had that feature been available at the time of the study. To examine the influence of this feature, optimization of a HT plan for a PMRT patient was performed with and without an objective of V_5Gy_ < 70% in the lungs using the same optimization procedure previously described. The value of V_5Gy_ was found to decrease from 93.4% to 85.5% when using utilizing the V_5Gy_ objective; however, dose to other structures correspondingly increased (for example, V_15Gy_ in the heart increased from 19.8% to 22.5%). Thus, we estimate that the use of low dose objectives during HT planning may slightly reduce the low dose volume, but would be unlikely to change the conclusions of this study given the wide margin by which VMAT plans reduced low dose volumes compared with HT (average lung V_5Gy_ of 66.2% *vs*. 89.8%).

Finally, delivering an adequate dose to the skin is critical during PMRT, as there is the substantial risk of recurrence in the chest wall [24]. Even with the use of thermoplastic bolus, substantial portions of the target volume are within 2 cm of the surface, and accurate dose calculation is challenging in this area, particularly with rotational delivery schemes. Using thermoluminescent dosimeters, our group previously reported on *in vivo* dose verification of skin doses from HT plans for PMRT as calculated by the TomoTherapy planning system [25]. For VMAT, the accuracy of superficial doses calculated by the Pinnacle treatment planning system has been documented for IMRT in other sites [26]. While preliminary *in vivo* verification measurements of skin doses from VMAT for PMRT patients in our clinic have shown similarly good agreement, future work may include a clinical protocol for systematic evaluation of *in vivo* doses from VMAT plans for PMRT, similar to our previous report on HT. Additional future studies may also include investigation of methods that reduce low dose spill of both VMAT and HT plans or the use of fixed-field IMRT in cases where neither VMAT nor HT are clinically available.

## Conclusions

VMAT is capable of producing treatment plans for PMRT that are dosimetrically and radiobiologically comparable to HT and can be delivered in approximately 66% less time. Currently, both modalities are used to treat PMRT at our clinic.

## Competing interests

This work was supported in part by a research agreement with Elekta Ltd. However, Elekta, Ltd., did not participate in the study design; in the collection, analysis, and interpretation of data; in the writing of the manuscript; or in the decision to submit the manuscript for publication.

## Authors’ contributions

GN constructed treatment plans and acquired the data with the assistance of JF and JG. MS identified patients and evaluated treatment plans. GN and JF analyzed the data. All authors participated in the design of the study, and preparation and approval of the final manuscript.
